# Analysis identifying minimal governing parameters for clinically accurate *in silico* fractional flow reserve

**DOI:** 10.3389/fmedt.2022.1034801

**Published:** 2022-12-06

**Authors:** Cyrus Tanade, S. James Chen, Jane A. Leopold, Amanda Randles

**Affiliations:** ^1^ Department of Biomedical Engineering, Duke University, Durham, NC, United States; ^2^ Department of Medicine, University of Colorado, Aurora, CO, United States; ^3^ Division of Cardiovascular Medicine, Brigham and Women’s Hospital, Boston, MA, United States

**Keywords:** fractional flow reserve, computational fluid dynamics, patient-specific modeling, sensitivity analysis, uncertainty quantication, Sobol analysis

## Abstract

**Background:**

Personalized hemodynamic models can accurately compute fractional flow reserve (FFR) from coronary angiograms and clinical measurements (FFR${}_{\mathrm{baseline}}$), but obtaining patient-specific data could be challenging and sometimes not feasible. Understanding which measurements need to be patient-tuned vs. patient-generalized would inform models with minimal inputs that could expedite data collection and simulation pipelines.

**Aims:**

To determine the minimum set of patient-specific inputs to compute FFR using invasive measurement of FFR (FFR${}_{\mathrm{invasive}}$) as gold standard.

**Materials and Methods:**

Personalized coronary geometries (N=50) were derived from patient coronary angiograms. A computational fluid dynamics framework, FFR${}_{\mathrm{baseline}}$, was parameterized with patient-specific inputs: coronary geometry, stenosis geometry, mean arterial pressure, cardiac output, heart rate, hematocrit, and distal pressure location. FFR${}_{\mathrm{baseline}}$ was validated against FFR${}_{\mathrm{invasive}}$ and used as the baseline to elucidate the impact of uncertainty on personalized inputs through global uncertainty analysis. FFR${}_{\mathrm{streamlined}}$ was created by only incorporating the most sensitive inputs and FFR${}_{\text{semi-streamlined}}$ additionally included patient-specific distal location.

**Results:**

FFR${}_{\mathrm{baseline}}$ was validated against FFR${}_{\mathrm{invasive}}$ via correlation (r=0.714, p<0.001), agreement (mean difference: 0.01±0.09), and diagnostic performance (sensitivity: 89.5%, specificity: 93.6%, PPV: 89.5%, NPV: 93.6%, AUC: 0.95). FFR${}_{\text{semi-streamlined}}$ provided identical diagnostic performance with FFR${}_{\mathrm{baseline}}$. Compared to FFR${}_{\mathrm{baseline}}$ vs. FFR${}_{\mathrm{invasive}}$, FFR${}_{\mathrm{streamlined}}$ vs. FFR${}_{\mathrm{invasive}}$ had decreased correlation (r=0.64, p<0.001), improved agreement (mean difference: 0.01±0.08), and comparable diagnostic performance (sensitivity: 79.0%, specificity: 90.3%, PPV: 83.3%, NPV: 87.5%, AUC: 0.90).

**Conclusion:**

Streamlined models could match the diagnostic performance of the baseline with a full gamut of patient-specific measurements. Capturing coronary hemodynamics depended most on accurate geometry reconstruction and cardiac output measurement.

## 1. Introduction

Computational blood flow models that minimize the number of patient-tuned parameters required to extract diagnostic phenomarkers accurately may circumvent clinical data acquisition challenges and help enable interventional planning. One particular application is coronary artery disease—a leading cause of death and disability worldwide, with 7 million deaths and 129 million disability-adjusted life years lost annually (1). Invasively-measured fractional flow reserve (FFR${}_{\mathrm{invasive}}$) is the gold standard for identifying atherosclerotic lesions requiring intervention (2, 3). In recent years, computational fluid dynamics (CFD) models that predict FFR have emerged. These models are either based on coronary angiography (4, 5) or computed tomography (6, 7). Some of these models have been used extensively for research, such as VIRTUHeart (8–12), or have been made available to market, namely FFR${}_{\mathrm{CT}}$ (HeartFlow, Mountain View, CA, USA) (13, 14), FFR${}_{\mathrm{angio}}$ (CathWorks, Kfar-saba, Israel) (15–17), CAAS-vFFR (Pie Medical, Maastricht, The Netherlands) (18, 19), and QFR (Medis Medical Imaging, Leiden, The Netherlands and Pulse Medical Technology Inc., Shanghai, China) (20–22). However, these models require many clinically-measured inputs to accurately capture the effect of stenoses. In theory, a CFD model incorporating a maximum number of patient-tuned inputs would calculate the most accurate FFR. Requiring large numbers of invasively-measured parameters is challenging, costly, and sometimes not feasible. CFD models incorporating extensive patient-specific measurements have limited use when patients lack the complete set of necessary parameters for flow simulation. The pervasiveness of missing data in electronic health records increases the prevalence of such cases (23–25). Contrary to intuition, requiring full patient-specificity, which includes personalizing the computational domain, physical properties of blood vessels, and boundary conditions that dictate flow, may not even be required to recover diagnostic phenomarkers accurately. A low-cost CFD model based on only a few patient-derived measurements could streamline costly clinical data acquisition pipelines without compromising the diagnostic performance of fully personalized models. Prior sensitivity analysis and uncertainty quantification studies have attempted to identify the key anatomic and physiologic parameters contributing to FFR, but these studies often rely on models that have not been validated against FFR${}_{\mathrm{invasive}}$ measurements and are limited by small cohort sizes (11, 26–29).

Prior studies have demonstrated that accurately capturing stenosis geometry (in terms of minimal luminal radius and stenosis length) through imaging and prescribing flow distribution down the coronary tree (27) are the most sensitive inputs. Controlling flow distribution throughout the coronary tree is a function of terminal branch geometry as determined by Murray’s Law, which may indicate that coronary anatomy is the overriding input (30). Ensuring accurate coronary anatomy could allow some leeway for the variance of other parameters. When patient-tuned values do not drastically deviate from patient averages, patient-generalized inputs could result in the same FFR calculation. As the focus has been on identifying sensitive inputs, it is unknown which parameters are insensitive to FFR and could be relegated to patient averages, or patient-generalized parameters, without sacrificing diagnostic performance (31). We hypothesized that on top of accurately segmenting the overall coronary tree, an accurate model with minimized inputs would require prescribing flow distribution parameters and capturing the geometric severity of stenoses on a per-patient level. In this work, we present a patient-specific CFD FFR model (FFR${}_{\mathrm{baseline}}$) and validated the model in a cohort of 50 patients. Sobol decomposition techniques were used to derive optimized, low-cost models (FFR${}_{\text{semi-streamlined}}$ and FFR${}_{\mathrm{streamlined}}$) with minimal patient-specific clinical inputs without sacrificing diagnostic performance and agreement compared to FFR${}_{\mathrm{invasive}}$.

## 2. Materials and methods

### 2.1. Patient data

**Figure 1:**
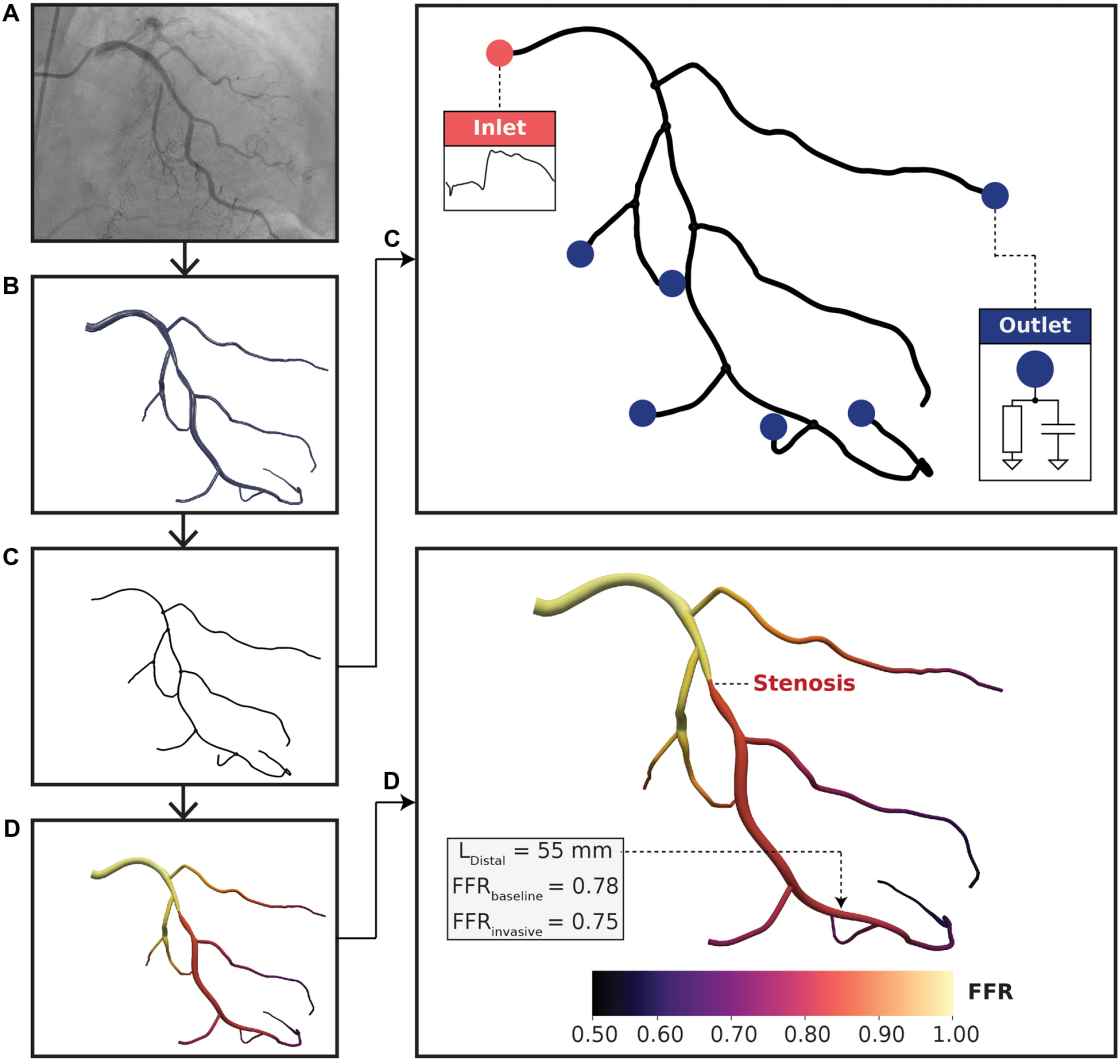
Computational fluid dynamics modeling pipeline. (**A**) Coronary angiograms were acquired for each patient. At least 4 or 2 standard orthogonal views were collected for left and right coronary trees, respectively. (**B**) A three-dimensional (3D) arterial tree model was semi-automatically reconstructed using a pair of coronary angiograms. (**C**) Vessel centerlines were extracted from the 3D reconstructed geometry for one-dimensional (1D) simulation. Pulsatile flow rate was used at the inlet boundary condition and 2-element Windkessel models were used at the outlet boundary conditions. The lumped parameter models consisted of resistance-compliance components, where resistances were related to terminal vessel anatomy. (**D**) FFR results mapped on a left coronary vessel. The distal location (L${}_{\mathrm{Distal}}$) was labeled by an expert physician and was situated 55 mm downstream to the distal-end of the stenosis. FFR${}_{\mathrm{baseline}}$ and FFR${}_{\mathrm{invasive}}$ resulted in the same FFR classification with minimal discrepancy.

This study did not involve human tissue samples, direct patient experimentation, or interaction. The protocol was approved by the Massachusetts General Brigham Institutional Review Board (IRB Protocol #2015P001084). The IRB did not require individual patients to sign informed consent since the study was not prospective and there was no patient interaction or intervention performed. Patient data, consisting of coronary angiograms and clinical measurements, were acquired from 50 patients who underwent a clinically indicated coronary angiogram and were found to have angiographically-documented coronary artery disease at Brigham and Women’s Hospital, Boston, MA, USA. Exclusion criteria were prior coronary artery bypass graft surgery, ST-elevation myocardial infarction, chronic total occlusion, and ostial lesions. The 50 patients were randomly selected. Angiograms included at least 4 standard orthogonal views of the left coronary circulation and 2 standard orthogonal views of the right coronary circulation (Figure 1A). Clinical measurements were collected during coronary angiography to inform personalized blood flow simulations, including aortic blood pressure, cardiac output, heart rate, and hematocrit. Routine FFR measurements were performed by administering intravenous adenosine (140mcg/kg/min×120s) to induce hyperemic conditions. A coronary guidewire pressure sensor (Volcano Corporation, San Diego, CA) was placed distal to the coronary stenosis for *in vivo* FFR computation. An experienced interventional cardiologist selected the location of distal pressure measurement, ranging 5–65 mm with respect to the distal-end of the stenosis. FFR≤0.80 was considered ischemic and FFR>0.80 was considered non-ischemic. The researchers performing the coronary reconstructions and CFD simulations were blinded to clinically measured FFR values until CFD validations were completed.

### 2.2. Coronary geometry reconstruction

Three-dimensional (3D) full coronary tree models (Figure 1B) were reconstructed from pairs of coronary angiograms using a semi-automated algorithm described in (32, 33). The algorithm first computed two-dimensional vessel centerlines with corresponding cross-sectional diameters semi-automatically on the pair of images. Afterward, a fully automated computation was used to generate the 3D coronary tree models in stereolithography (STL). Reconstructions were validated topologically and anatomically by an expert interventional cardiologist using ImageJ v1.52k (NIH, Bethesda, MD, USA) via comparing the minimal luminal diameter of the stenotic lesion segment in 3D models with the minimal diameter of the same diseased arterial segment in angiograms, which were additional cine runs acquired different from the pair of images (or the 3rd view) as used for 3D reconstruction. All identifiable (>1 mm) main and side branch vessels were reconstructed from 2D coronary angiography data. To create one-dimensional (1D) geometries (Figure 1C), vessel centerlines with corresponding hydraulic diameters were extracted from the reconstructed STL models using Mimics (Materialise, Leuven, BE). Vessel lengths were computed from centerline outputs at a resolution of 100 micrometers. The centerline data was validated by comparing the minimal luminal diameter between the 1D model with physician-measured ground truths on angiograms. These 1D full coronary tree models were used as inputs to personalized CFD simulations. Coronary anatomy was used to define the computational domain and was required to be reconstructed accurately on a per-patient level.

### 2.3. 1D computational model

#### 2.3.1. Model assumptions

Blood was modeled as an incompressible Newtonian fluid with ρ=1,060 kg/m${}^{3}$. Dynamic viscosity was computed per-patient using an empirical relationship between viscosity and hematocrit from (34):

(1) $$\mu =\frac{\mu_{0}}{1-\phi }$$

where μ is the dynamic viscosity of blood, $\mu_{0}$ is the dynamic viscosity of plasma, and ϕ is hematocrit. We assumed a constant plasma hematocrit of 1.2 cP (4, 5, 34). The 1D blood flow simulator inherently uses elastic walls, but we enforced quasi-rigid walls. We noted area deformations of 0.78% when averaged across all cases, all vessels, and over all time points. The area deformations here were comparable to other 1D models (35) that enforced quasi-rigid walls.

#### 2.3.2. Mathematical formulation

We used a 1D blood flow model described in (36, 37). The 1D blood flow simulator was based on the following governing equations:

(2) $$\frac{\partial A}{\partial t}+\frac{\partial Q}{\partial x}=0$$

(3) $$\frac{\partial Q}{\partial t}+\frac{\partial }{\partial x}\left(\alpha \frac{Q^{2}}{A}\right)+\frac{A}{\rho }\frac{\partial P}{\partial x}=-C_{\;f}\frac{Q}{A}$$

where A is vessel cross-sectional area, Q is flow rate, P is pressure, ρ is density of blood, α describes the velocity profile, and $C_{\;f}=22\pi \mu$ is a frictional term. α and $C_{\;f}$ were estimated from experimental data that have been used in 1D models (36–39). P and A are related by the following constitutive equation:

(4) $$P=P_{\mathrm{ext}}+\beta (\sqrt{A}-\sqrt{A_{0}}),\;\beta =\frac{\sqrt{\pi }hE}{(1-\nu^{2})A_{0}}$$

where $P_{\mathrm{ext}}$ is external pressure exerted on vessels and $A_{0}$ is the undeformed cross-sectional area when $P=P_{\mathrm{ext}}$. β describes arterial stiffness and is a function of $A_{0}$, wall thickness (h=0.945 mm) (40), elastic modulus (E=1.41 MPa) (41), and Poisson’s ratio (ν=0.5) (42). The conservation of mass (Equation 2), conservation of momentum (Equation 3), and pressure-area constitutive relationship (Equation 4) were solved using a MacCormack finite difference scheme.

To model pressure drop across stenoses, we coupled the 1D model with an explicit pressure loss term:

(5) $$\Delta P_{s}=\frac{\mu K_{v}}{2\pi r_{u}^{3}}Q+\frac{\rho K_{t}}{2A_{u}^{2}}{\left(\frac{A_{u}}{A_{s}}-1\right)}^{2}|Q|Q+\frac{\rho K_{u}L_{s}}{A_{u}}\frac{\partial Q}{\partial t}$$

where $\Delta P_{s}$ is pressure drop across a focal stenosis, $r_{u}$ is radius of an unstenosed artery, $A_{u}$ is cross-sectional area of an unstenosed artery, $A_{s}$ is cross-sectional area of a stenosed artery, and $L_{s}$ is stenosis length. The stenosis model (**Equation 5**) was coupled to the 1D governing equations via the continuity of total pressure. The anatomical position of stenoses were labelled by expert interventional cardiologists. $r_{u}$ was estimated from physician labeled stenosis degree and minimal luminal radius ($r_{s}$), using the following expression:

(6) $$\text{Stenosis degree}=\left(1-\frac{r_{s}}{r_{u}}\right)\times 100\text{\%}$$

$A_{s}$ was parameterized using the minimal luminal diameter. $K_{v}$, $K_{t}$, and $K_{u}$ are viscous, “turbulent,” and inertial coefficients, respectively. The “turbulent” term reflects non-linear effects of converging or diverging flow patterns, for example swirling or chaotic flow downstream of the distal end of a stenosis. These coefficients were parameterized as $K_{v}=32(0.83L_{s}+1.64D_{s})\;\times \;\;\;\;\;$ $(A_{u}/A_{s}{)}^{2}/D_{u}$, $K_{t}=1.52$, and $K_{u}=1.2$ based on (36). $D_{u}$ and $D_{s}$ are unstenosed and stenosed arterial diameters, respectively.

#### 2.3.3. Personalized boundary conditions

To tune the boundary conditions to each patient, a pulsatile flow rate waveform was incorporated at the inlet and 2-element Windkessel models at the outlets (Figure 1C). Left and right coronary waveforms were derived from (43) and scaled to a patient-specific level using clinically measured cardiac output, heart rate, and flow dominance at resting state. Hyperemia was simulated by scaling up the flow rate based on empirical observations, where the left and right circulations were scaled up by 4x and 3x, respectively (44).

A 2-element Windkessel model, consisting of peripheral resistance ($R_{\;p}$) and compliance (C), was applied to the ends of each terminal vessel to account for the effect of microvascular hemodynamics (45):

(7) $$Q=\frac{P}{R_{\;p}}+C\frac{dP}{dt}$$

C was assumed to be constant at 9 $\text{μ}{\mathrm{cm}}^{4}\;s^{2}\;g^{-1}$ based on patient averages from (36). $R_{\;p}$ was distributed among the terminal branches using resistance-radius relationships commonly used for coronary simulations (4, 5, 14, 46):

(8) $$R_{i}=P_{\mathrm{mean}}\cdot \frac{\sum_{\;j=1}^{N_{\mathrm{terminal}}}r_{\;j}^{3}}{Q_{\mathrm{ostial}}r_{i}^{3}}$$

where $R_{i}$ is the peripheral resistance at each terminal branch, $P_{\mathrm{mean}}$ is mean arterial pressure, $Q_{\mathrm{ostial}}$ is flow rate at the ostium, r is the average terminal branch radius, and $N_{\mathrm{terminal}}$ is the number of terminal branches. Resting state mean arterial pressure and inlet flow rate were both obtained from clinical measurements and terminal branch radii were computed via 1D vessel centerlines. Specifically, the ostial flow rate was determined as a fraction of cardiac output via flow dominance (4, 5). Terminal resistances were scaled down by a factor of 0.22x (4, 5, 14, 44) from the resting state to estimate hyperemia.

#### 2.3.4. Model convergence

Blood flow was simulated for 20 cardiac cycles based on temporal convergence tests, and the last cardiac cycle was used for analysis (Figure 1D). The grid spacing, or Euclidean distance between fluid points, was set to 500 micrometers based on grid invariance tests. The time step was $10^{-5}$ s to satisfy the Courant-Friedrichs-Lewy condition. The convergence criterion was $L_{2}$ error $<10^{-3}$ based on similar CFD studies (4, 5, 47–50). The metric of interest was time-averaged pressure at the distal location. Pressure at the distal location was selected because this was used to compute FFR. Temporal and spatial convergence data could be found in Supplementary Figure S1 and Table S1, respectively.

### 2.4. Defining the streamlined model for calculating fractional flow reserve

Understanding which clinical inputs could be relegated to patient averages was the first step to developing a streamlined computational FFR framework. We used global uncertainty analysis to elucidate which raw clinical measurements were critical to computing FFR accurately, as defined by a validated baseline model with all parameters derived from patient data, FFR${}_{\mathrm{baseline}}$.

#### 2.4.1. Mathematical basis of global uncertainty quantification

We employed variance-based global uncertainty quantification techniques as explained by Eck et al. (51). Global was selected over local uncertainty quantification to most uniformly sample the multi-dimensional parameter space and capture non-additive, non-monotonic, and non-linear effects and interactions between inputs (51). Global uncertainty quantification enabled assessment of the individual contribution of input parameters to the overall variance in FFR as well as the interaction between input parameters. Sobol indices were used to quantify the impact of clinical inputs on FFR:

(9) $$S_{i}=\frac{V[E[Y|Z_{i}]]}{V[Y]}$$

(10) $$S_{ij}=\frac{V[E[Y|Z_{i},Z_{j}]]}{V[Y]}$$

(11) $$S_{Ti}=1-\frac{V[E[Y|Z_{-i}]]}{V[Y]}$$

where $S_{i}$ captures the main effect of input parameter $Z_{i}$ (neglecting interaction between inputs) to the total variance V[Y], $S_{ij}$ quantifies the effect of interaction between inputs $Z_{i}$ and $Z_{\;j}$, and $S_{Ti}$ quantifies the sum total of main and interaction effects. The $V[E[Y|Z_{i}]]$ term is the variance of the expected value of output Y given a fixed value of input parameter $Z_{i}$. $Z_{-i}$ is a set of all input parameters excluding $Z_{i}$. When $S_{i}\approx S_{Ti}$, interaction effects are negligible, suggesting that main effects drove the variance in Y.

#### 2.4.2. Parameterizing clinical inputs

Incorporating a complete set of inputs could be injudicious if many parameters do not significantly contribute to FFR, which would in effect only enlarge the sample space needlessly. The raw inputs to define personalized blood flow simulations included mean arterial pressure, cardiac output, coronary geometry, heart rate, stenosis anatomy, hematocrit, and distal location. We considered stenosis anatomy separately from coronary geometry. Explicit pressure drop terms were required to accurately evaluate ischemic burden, and these terms were parameterized via stenosis geometry: stenosis length and stenosis radius at the minimum luminal diameter (30, 31). Cardiac output was used to parameterize the inlet flow rate waveform and peripheral resistance at the outlets. To prevent diluting the parameter space with insignificant parameters, the raw inputs were narrowed to mean arterial pressure, cardiac output, stenosis degree, and distal location based on what has been shown to be significant from prior works (27, 31). As inlet flow rate was found to be insignificant in the literature (27, 31, 52), we only evaluated cardiac output as it pertained to peripheral resistance and relegated cardiac output as it pertained to inlet flow rate as patient-generalized. The importance of distal location has been discussed in (53) and was also considered potentially significant. From this point forward, we define distal location as the anatomic location of pressure sampling distal to the stenosis. Supplementary Figure S2 summarizes the raw inputs we investigated within the context of the CFD framework.

The uncertainty bounds, or range of allowed uncertainty, for each clinical input was either derived from literature or estimated from the patient population (Table 1). All uncertainty bounds in clinical inputs were modeled as normal distributions (51). Patient-specific cardiac output and mean arterial pressure were varied by multiplying with scaling factors, modeled as normal distributions with means of unity and standard deviations from literature-derived coefficients of variations (27, 54). The error bound in stenosis degree was modeled to reflect the worst-case inter-observer variability. The difference in stenosis degrees measured by an interventional cardiologist and a researcher, with measurements blinded to each other, was used to parameterize a normal distribution of uncertainty. The mean was fixed to zero to reflect the case when both observers agreed perfectly. The standard deviation directly quantified the error bound and was added to the baseline stenosis degree to probe uncertainty. For distal location, we estimated a coefficient of variation (CV=0.117; see Supplementary Figure S3) and mean (30 mm) based on the patient population and clinical recommendations (55). The values sampled from the resulting normal distribution were used as the distal location to compute FFR in the global uncertainty analysis.

**Table 1:** Input parameter bounds to study the impact of patient-specificity on FFR.

Clinical input	Type	Distribution
Distal location (mm)	Value	N(30.0,3.5)
Cardiac output (%)	Factor	N(1,0.153)
Stenosis degree (%)	Addition	N(0,16.9)
Mean arterial pressure (%)	Factor	N(1,0.056)

Normal distributions denoted as N(μ,σ).

#### 2.4.3. Evaluating the relative contribution of clinical inputs to fractional flow reserve

After defining the parameters and range of uncertainties to explore, we needed to perturb FFR${}_{\mathrm{baseline}}$ models to compute Sobol indices and evaluate the relative contribution to FFR. The normal distributions in Table 1 were sampled according to a second order Saltelli sequence. This sampling technique has been shown to minimize error rates in estimating the Sobol indices (56, 57). Sobol indices were considered as converged when the bootstrapped 95% confidence interval width of the main and total effects were smaller than 10% of the maximum Sobol index for each parameter (58). FFR was computed for every combination of the four clinical inputs from the Saltelli sequence. Sobol indices were computed on the aggregate of all FFR values to relate the impact of uncertainty in clinical inputs to the resulting FFR value. Based on tests, over 1 million simulations were required to achieve converged Sobol indices. A high number of simulations were also noted by Eck et al. (51) to obtain convergence. We used an embarassingly parallel scheme to run multiple simulations simultaneously. The simulations were completed with wall clock duration of two weeks on 32 compute nodes. Sobol indices exceeding a threshold of 0.05 were considered significant (51).

#### 2.4.4. Establishing and evaluating FFR${}_{\mathrm{streamlined}}$

**Figure 2:**
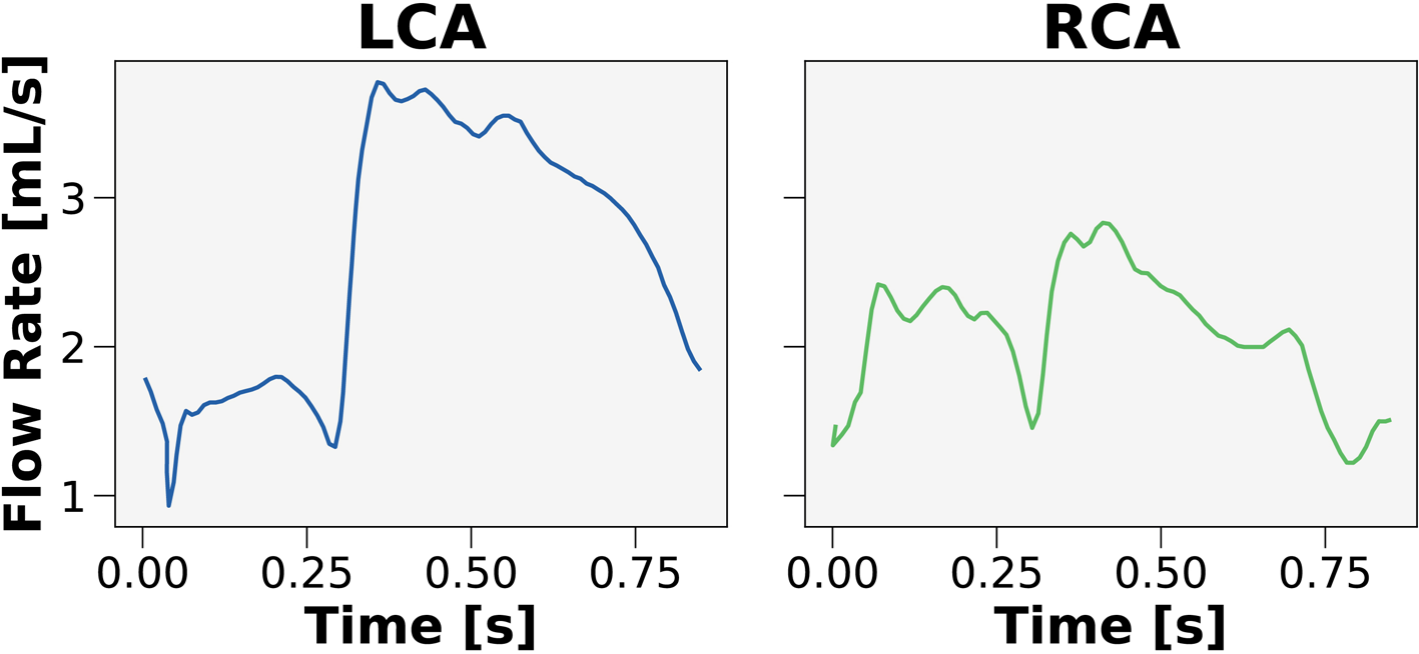
Canonical waveforms for the left and right coronary circulation. Hyperemic pulsatile flow rate waveforms were used as inputs to the streamlined model for the left (LCA) and right (RCA) coronary arteries. The waveforms were generalized over the 50 patients in the cohort.

The Sobol indices were used to identify which clinical inputs were most important and inform on streamlining the CFD framework. Patient-generalized values were used for parameters that had relatively little contribution to FFR. These values were computed by taking the average over the entire cohort of patients to be used as inputs in the streamlined model. One parameter that could be patient-generalized from the get-go was inlet flow rate waveform. As prior studies (27, 31, 52) demonstrated that the inlet flow rate waveform contributed minimally to FFR, two canonical hyperemic waveforms were created (Figure 2), one for the left coronary artery (LCA) and one for the right coronary artery (RCA).

After identifying all the patient-generalized parameters via global uncertainty quantification, the streamlined model was compared to the baseline framework and clinical ground-truths. Correlation between streamlined models (FFR${}_{\text{semi-streamlined}}$ and FFR${}_{\mathrm{streamlined}}$) with FFR${}_{\mathrm{baseline}}$ and FFR${}_{\mathrm{invasive}}$ was determined using a least-squares linear regression. Bland-Altman analysis was used to evaluate the mean differences between streamlined models with FFR${}_{\mathrm{baseline}}$ and FFR${}_{\mathrm{invasive}}$. We also evaluated ability of streamlined models to classify ischemic vs. non-ischemic stenoses identified by FFR${}_{\mathrm{invasive}}$, using metrics such as sensitivity, specificity, positive predictive value, negative predictive value, and overall accuracy. Finally, receiver-operating characteristics (ROC) curves were used to compute an area under the curve (AUC) and to recover the 0.80 ischemic threshold to evaluate bias. An unbiased model would recover the 0.80 threshold and trade-off the true positive and false positive rate. The optimum threshold from an ROC curve was taken as the point that maximizes sensitivity (or true positive rate) and minimizes 1-specificity (or false positive rate).

#### 2.4.5. Determining if relative contribution is generalizable across patients

As a secondary endpoint to this work, we evaluated whether Sobol indices varied across patients. Previous works have conventionally aggregated the Sobol indices over all patients in the cohort, but the relative importance of each input parameter to FFR may not generalize across differing patient anatomy and physiology (11, 27, 31). To this end, we performed a global repeated measures analysis of variance (ANOVA), where anatomic and hemodynamic variations subdivided the Sobol indices. Specifically, anatomy was subdivided by LCA and RCA. We considered two hemodynamic variations. Ischemia-inducing disease was considered at the clinical threshold of 0.80. Patients were stratified using this classification to probe how the impact of patient-specificity could differ between functionally significant and insignificant stenoses. As a positive control, we also incorporated the grey-zone FFR—a range of FFR values, between 0.75–0.85, traditionally known to be of uncertain ischemic burden (59). Subdividing by grey-zone provided a baseline variance to compare with coronary vessel and ischemia classification subgroups. Successive factorial ANOVAs were performed to reveal the most important subdivisions in the parameter space using variance.

### 2.5. Statistical analysis

The Kolmogorov-Smirnov test was used to ensure that the data followed the central limit theorem. Predicted FFR was evaluated against the clinical ground-truth via least-squares correlation and Bland-Altman analysis. ANOVA and post hoc tests were performed on JMP Pro 16 (JMP Statistical Discovery LLC, Cary, NC, USA). Diagnostic performance metrics between models were compared using paired t-test or Wilcoxon signed-rank test. A value of p<0.05 was considered significant.

## 3. Results

### 3.1. Patient and clinical characteristics

A retrospective cohort of 50 patients was created from adults with angiographically documented coronary artery disease, involving at least one vessel with FFR${}_{\mathrm{invasive}}$ measurement between January 1, 2016 and August 1, 2018. Patient characteristics are highlighted in Table 2. A total of 69.4% of patients were male. The mean age was 66.5 years. 2.0% had hypertension, 8.2% had hypercholesterolemia, and 32.7% had diabetes mellitus. The mean left ventricular ejection fraction was 58.2%. The most common medications at the time of cardiac catheterization were lipid lowering agents (93.9%), aspirin (87.8%), and ACEi/ARB (59.2%). Stenosis characteristics are presented in Table 3. 79.6% of stenoses were right dominant, 12.2% were left dominant, and 8.2% were co-dominant circulations. The mean stenosis degree was 55.6%. Stenosis that underwent intervention were found in the left anterior descending artery (53.1%), left circumflex artery (20.4%), and right coronary artery (26.5%). The majority of stenoses were concentric (65.3%).

**Table 2:** Aggregated characteristics of patients (N=50).

Age (years)	66.5±9.5
Female (%)	30.6
Hypertension (%)	2.0
Hypercholesterolemia (%)	8.2
Diabetes mellitus (%)	
Type I	8.2
Type II	24.5
Tobacco use (%)	
Current	4.1
Former	36.7
Never	59.2
Prior MI (%)	44.9
Prior PCI (%)	46.9
Prior CABG (%)	0.0
Congestive heart failure (%)	34.7
Peripheral arterial disease (%)	26.5
Chronic kidney disease (%)	28.6
Weight (kg)	87.8±20.6
Systolic blood pressure* (mmHg)	125.8±25.8
Diastolic blood pressure* (mmHg)	67.1±12.7
Heart rate* (bpm)	70.8±13.7
Cardiac output* (L/min)	4.5±1.5
Left ventricular ejection fraction (%)	58.2±10.8
Medications (%)	
Aspirin	87.8
P2Y12 inhibitors	38.8
Anticoagulants	26.5
Lipid lowering agents	93.9
ACEi/ARB	59.2
Nitrates	20.4

Values are mean±SD or n (%). ${}^{*}$Values are in resting state conditions. MI, myocardial infarction; PCI, percutaneous coronary intervention; CABG, coronary artery bypass graft.

**Table 3:** Aggregated characteristics of vessels (N=50).

Vessel dominance (%)	
Right	79.6
Left	12.2
Co-dominant	8.2
SYNTAX score (%)	
Low	2.0
Medium	12.2
High	85.7
Invasive FFR vessel (%)	
LAD	53.1
LCx	20.4
RCA	26.5
Minimal luminal diameter (mm)	1.4±0.4
Minimal luminal area (mm${}^{2}$)	1.7±0.9
Stenosis degree (%)	55.6±17.2
Plaque Eccentricity (%)	
Concentric	65.3
Eccentric	34.7
Calcified (%)	44.9
Tortuous (%)	10.2
Thrombus (%)	8.2
Aneurysm (%)	2.0

Values are mean±SD or n (%). Invasive FFR, fractional flow reserve; LAD, left anterior descending artery; LCx, left circumflex artery; RCA, right coronary artery.

### 3.2. Patient-specific 1D coronary models agree with clinical measurements

**Figure 3:**
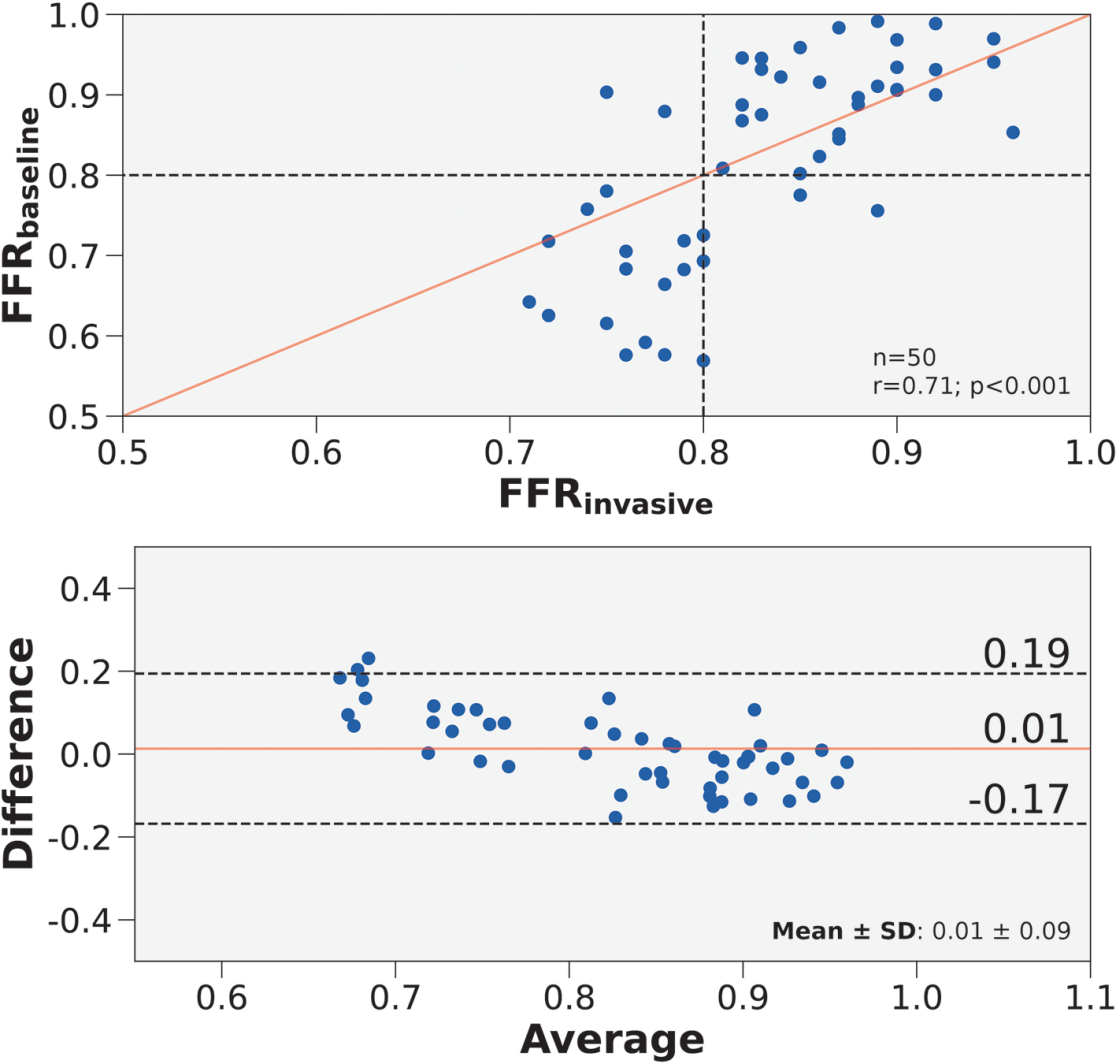
Correlation and agreement of FFR${}_{\mathrm{baseline}}$ compared to FFR${}_{\mathrm{invasive}}$. (Top) Scatter plot of FFR${}_{\mathrm{baseline}}$ and FFR${}_{\mathrm{invasive}}$ for 50 vessels. The number of stenoses and Pearson’s r are presented in the lower right hand corner. Interrupted lines represent the 0.80 ischemic threshold and the solid red line represents ideal correlation. (Bottom) Bland-Altman plot displaying the mean difference between FFR${}_{\mathrm{baseline}}$ and FFR${}_{\mathrm{invasive}}$ for 50 vessels. The mean difference and standard deviation are presented in the lower right corner. Black interrupted lines indicate the upper and lower limits of agreement (σ: ±1.96) and the solid red line indicates mean difference.

The first step in developing a streamlined framework was to validate a baseline CFD framework with full patient-tuned inputs—representing the best-case scenario. The baseline framework, FFR${}_{\mathrm{baseline}}$, was validated in 50 patients who had angiographically documented coronary artery disease. We validated FFR${}_{\mathrm{baseline}}$ against FFR${}_{\mathrm{invasive}}$. The correlation coefficient was 0.71 (p<0.0001) and the mean difference was 0.01±0.09 (Figure 3). Diagnostic performance of FFR${}_{\mathrm{baseline}}$ to discern ischemic stenoses are summarized in Table 5. The sensitivity was 89.5% (95% CI: 66.9–98.7%), specificity was 93.6% (95% CI: 78.6–99.2%), and overall accuracy was 92.0% (95% CI: 80.8–97.8%). Furthermore, the positive predictive value was 89.5% (95% CI: 68.8–97.0%) and the negative predictive value was 93.6% (95% CI: 79.6–98.2%).

### 3.3. Cardiac output and stenosis degree contributed most to fractional flow reserve

**Figure 4:**
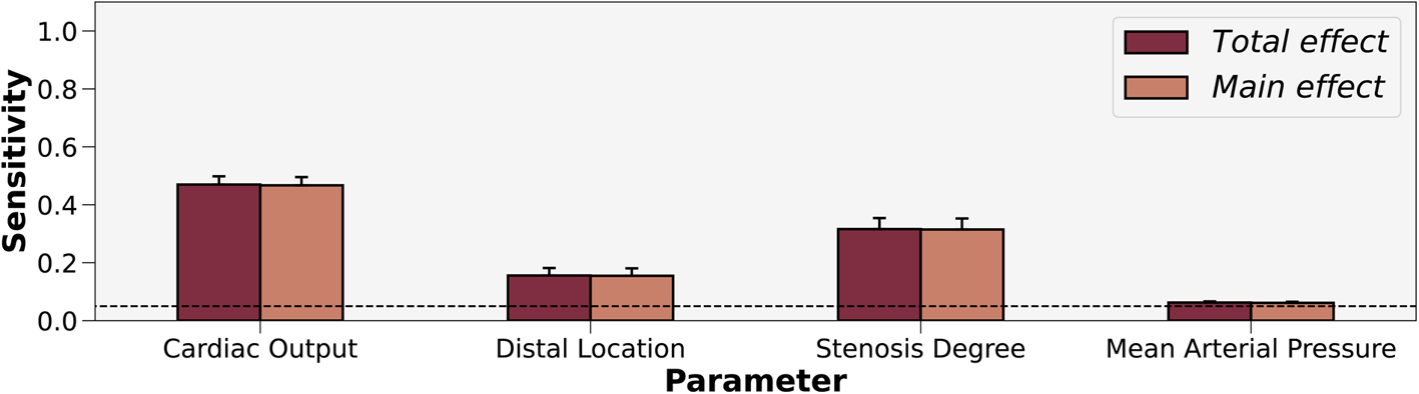
Sobol sensitivity indices of patient-tuned parameters on FFR. Total and main effects are displayed for cardiac output, distal location, stenosis degree, and mean arterial pressure. Cardiac output and stenosis degree contribute most to the variance in FFR. The horizontal interrupted line at 0.05 shows the threshold for sensitivity.

When aggregating all 50 patients, the uncertainty analysis (Figure 4) indicated that cardiac output and stenosis degree contributed most to the variance in FFR. Distal location and mean arterial pressure also exceeded the threshold for sensitivity, but contributed less to the variance in FFR than cardiac output and stenosis degree. While distal location exceeded the threshold for significance, Sobol indices are relative metrics and the effect sizes of cardiac output and stenosis degree exceeded distal location. To minimize the number of parameters, we created two streamlined models. FFR${}_{\text{semi-streamlined}}$ incorporated patient-generalized mean arterial pressure, and patient-specific cardiac output, stenosis degree, and distal location. FFR${}_{\mathrm{streamlined}}$ used patient-generalized distal location and mean arterial pressure, and patient-specific cardiac output and stenosis degree. The total and main effects were not statistically different, which meant that interaction effects between input parameters were insignificant.

### 3.4. Streamlined models maintain diagnostic performance

To test our findings from the aggregated Sobol indices, we parameterized the streamlined models with patient-generalized mean arterial pressure (87.3 mmHg), heart rate (70.8 bpm), hematocrit (39.2%), and ostial diameter (3.9 mm) (Table 4). With an average hematocrit of 39.2%, the average dynamic viscosity was 1.97 cP, which was comparable to viscosities used in other works at hyperemic state (4, 5). Patient-generalized cardiac output (4.5 L/min) was used to derive inlet flow waveforms, but patient-specific cardiac output was used to compute peripheral resistance. A patient-generalized distal location of 30 mm was used for FFR${}_{\text{semi-streamlined}}$.

**Table 4:** Patient-generalized clinical inputs.

Mean arterial pressure (mmHg)	87.3
Heart rate (bpm)	70.8
Cardiac output (L/min)	4.5
Hematocrit (%)	39.2
Ostial diameter (mm)	3.9

Clinical inputs were averaged over 50 patients and used to parameterize the streamlined model. Mean arterial pressure, heart rate, and cardiac output are in resting state conditions. Cardiac output was patient-generalized only as it pertained to inlet flow rate. Ostial diameter was obtained through coronary geometry reconstruction.

**Figure 5:**
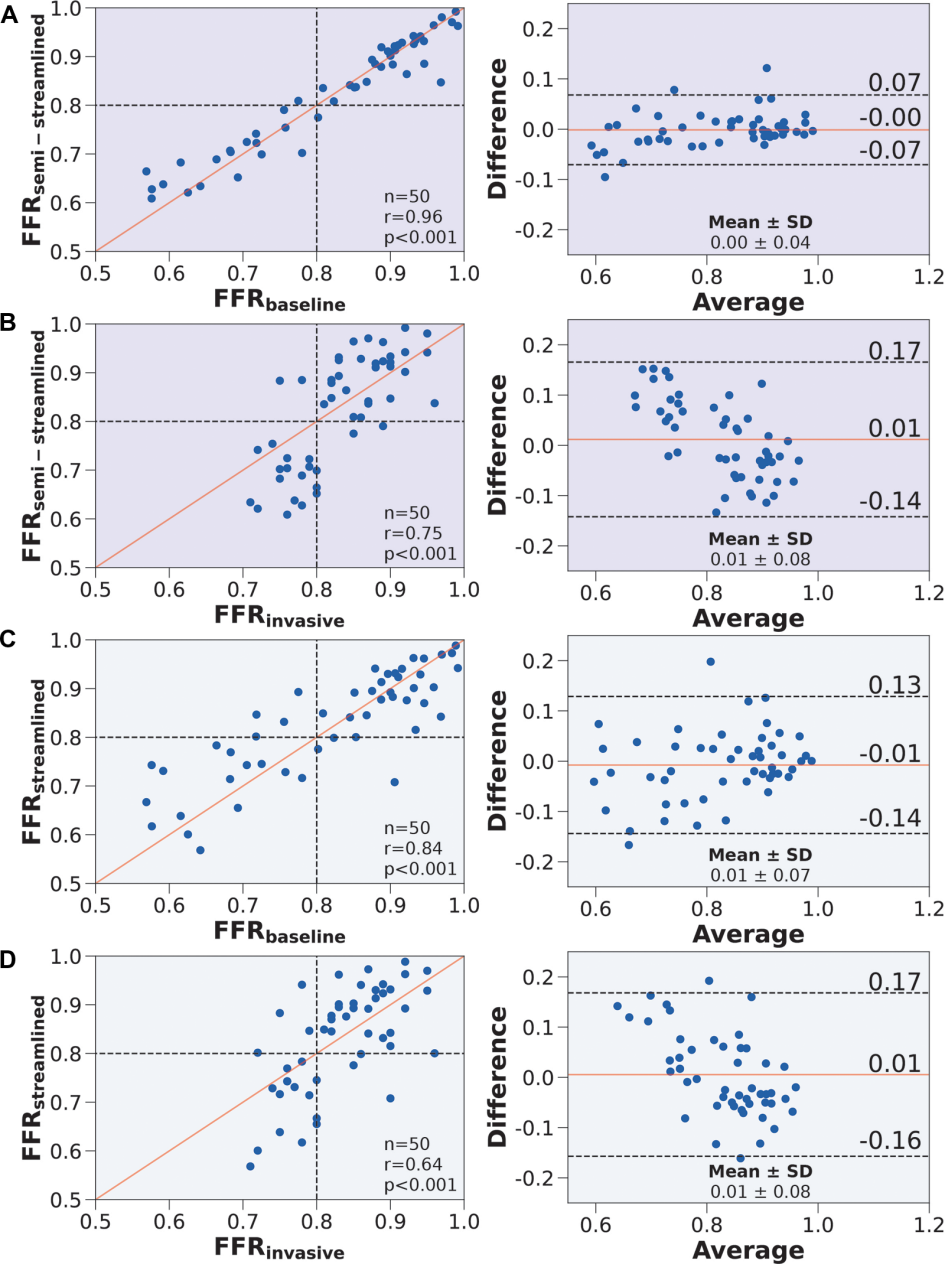
Correlation and agreement comparing FFR${}_{\text{semi-streamlined}}$, FFR${}_{\mathrm{streamlined}}$, FFR${}_{\mathrm{baseline}}$, and FFR${}_{\mathrm{invasive}}$. (Left) Scatter plots. The number of stenoses and Pearson’s r are presented in the lower right hand corner. Interrupted lines represent the 0.80 ischemic threshold and the solid red line represents ideal correlation. (Right) Bland-Altman plots. The mean difference and standard deviation are presented in the lower right corner. Black interrupted lines indicate the upper and lower limits of agreement (σ: ±1.96) and the solid red line indicates mean difference. We compared (**A**) FFR${}_{\text{semi-streamlined}}$ to FFR${}_{\mathrm{baseline}}$, (**B**) FFR${}_{\text{semi-streamlined}}$ to FFR${}_{\mathrm{invasive}}$, (**C**) FFR${}_{\mathrm{streamlined}}$ to FFR${}_{\mathrm{baseline}}$, and (**D**) FFR${}_{\mathrm{streamlined}}$ to FFR${}_{\mathrm{invasive}}$.

FFR${}_{\text{semi-streamlined}}$ compared well against FFR${}_{\mathrm{baseline}}$ in terms of correlation (r=0.96, p<0.001) and agreement (mean difference=0.00±0.04) (Figure 5A). Compared to FFR${}_{\mathrm{baseline}}$ vs. FFR${}_{\mathrm{invasive}}$, FFR${}_{\text{semi-streamlined}}$ vs. FFR${}_{\mathrm{invasive}}$ had slightly improved correlation (r=0.75, p<0.001) and agreement (mean difference=0.01±0.08) (Figure 5B). FFR${}_{\mathrm{streamlined}}$ compared well against FFR${}_{\mathrm{baseline}}$ in terms of correlation (r=0.84, p<0.001) and agreement (mean difference=0.01±0.07) (Figure 5C). Compared to FFR${}_{\mathrm{baseline}}$ vs. FFR${}_{\mathrm{invasive}}$, FFR${}_{\mathrm{streamlined}}$ vs. FFR${}_{\mathrm{invasive}}$ had a decrease in correlation (r=0.64, p<0.001) but a slight improvement in agreement (mean difference=0.01±0.08) (Figure 5D). The average percentage discrepancy compared to FFR${}_{\mathrm{baseline}}$ was 3.3% for FFR${}_{\text{semi-streamlined}}$ and 5.7% for FFR${}_{\mathrm{streamlined}}$.

**Figure 6:**
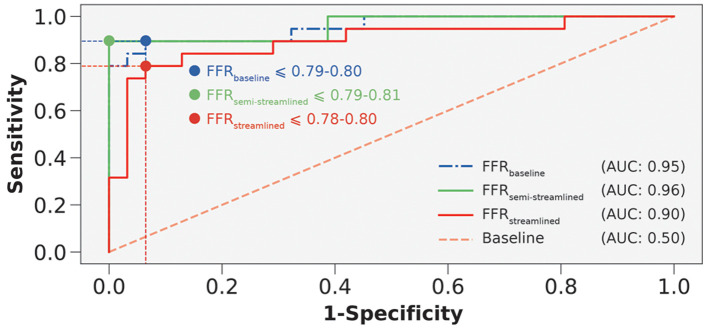
Receiver-operating characteristics curves to compare FFR${}_{\text{semi-streamlined}}$, FFR${}_{\mathrm{streamlined}}$, and FFR${}_{\mathrm{baseline}}$. AUC is area under the curve. Interrupted vertical and horizontal lines indicate the sensitivity and 1-specificity values that correspond to the optimum threshold. Blue, green, and red colored circles represent the threshold overlaid on the receiver-operating characteristics curves.

In terms of diagnostic performance to identify ischemic stenoses, the sensitivity was 89.5% (95% CI: 66.9–98.7%), specificity was 93.6% (95% CI: 78.6–99.2%), and overall accuracy was 92.0% (95% CI: 80.8–97.8%) for FFR${}_{\text{semi-streamlined}}$, which was identical to FFR${}_{\mathrm{baseline}}$. As for FFR${}_{\mathrm{streamlined}}$, the sensitivity was 79.0% (95% CI: 54.4–94.0%), specificity was 90.3% (95% CI: 74.3–98.0%), and overall accuracy was 86.0% (95% CI: 73.3–94.2%). To compare between the models (Table 5), we applied paired Wilcoxon signed-ranked tests with Holm-Bonferroni correction. The diagnostic metrics were not statistically significant for FFR${}_{\mathrm{streamlined}}$ vs. FFR${}_{\mathrm{baseline}}$ (p=0.125) and the diagnostic metrics were identical between FFR${}_{\text{semi-streamlined}}$ vs. FFR${}_{\mathrm{baseline}}$. We further validated the streamlined models by evaluating AUC and recovering the ischemic threshold (Figure 6). The idealized case, FFR${}_{\mathrm{baseline}}$, had an AUC of 0.95, and the ischemic threshold was recovered to be 0.79–0.80. FFR${}_{\text{semi-streamlined}}$ had an AUC of 0.96 and the ischemic threshold ranged between 0.79–0.81. FFR${}_{\mathrm{streamlined}}$ had an AUC of 0.90 and the ischemic threshold ranged between 0.78–0.80.

**Table 5:** Diagnostic performance of FFR${}_{\mathrm{streamlined}}$, FFR${}_{\text{semi-streamlined}}$, and FFR${}_{\mathrm{baseline}}$ to detect ischemic stenoses at the clinical threshold of 0.80.

Metric	FFR${}_{\mathrm{streamlined}}$	FFR${}_{\text{semi-streamlined}}$	FFR${}_{\mathrm{baseline}}$
Sensitivity	79.0 (54.4–94.0)	89.5 (66.9–98.7)	89.5 (66.9–98.7)
Specificity	90.3 (74.3–98.0)	93.6 (78.6–99.2)	93.6 (78.6–99.2)
PPV	83.3 (62.5–93.8)	89.5 (68.8–97.0)	89.5 (68.8–97.0)
NPV	87.5 (74.4–94.4)	93.6 (79.6–98.2)	93.6 (79.6–98.2)
Overall accuracy	86.0 (73.3–94.2)	92.0 (80.8–97.8)	92.0 (80.8–97.8)

### 3.5. Relative contribution of clinical inputs influenced by anatomy

As a secondary endpoint, we also evaluated whether Sobol indices could be generalized across patients. Using global ANOVA, grey-zone (p=0.0356), anatomy (p=0.0357), and the repeated measures of the Sobol indices (p<0.0001) were statistically significant main effects (Supplementary Table S2). The interaction effect of the repeated measures with anatomy (p=0.0293) was significant. Since the repeated measures captured most of the effect size and contributed to the significant interaction effect, we subdivided the clinical inputs and performed a factorial ANOVA. Distal location was found to have no significant effects (Supplementary Table S3). Anatomy had a significant main effect for cardiac output (p=0.0279) (Supplementary Table S4), stenosis degree (p=0.0025) (Supplementary Table S5), and mean arterial pressure (p=0.0266) (Supplementary Table S6). Since the contribution of mean arterial pressure to FFR was the least in the aggregated global uncertainty quantification results (Figure 4), we focused on elucidating how cardiac output and stenosis degree varied by anatomy via post hoc analysis.

**Figure 7:**
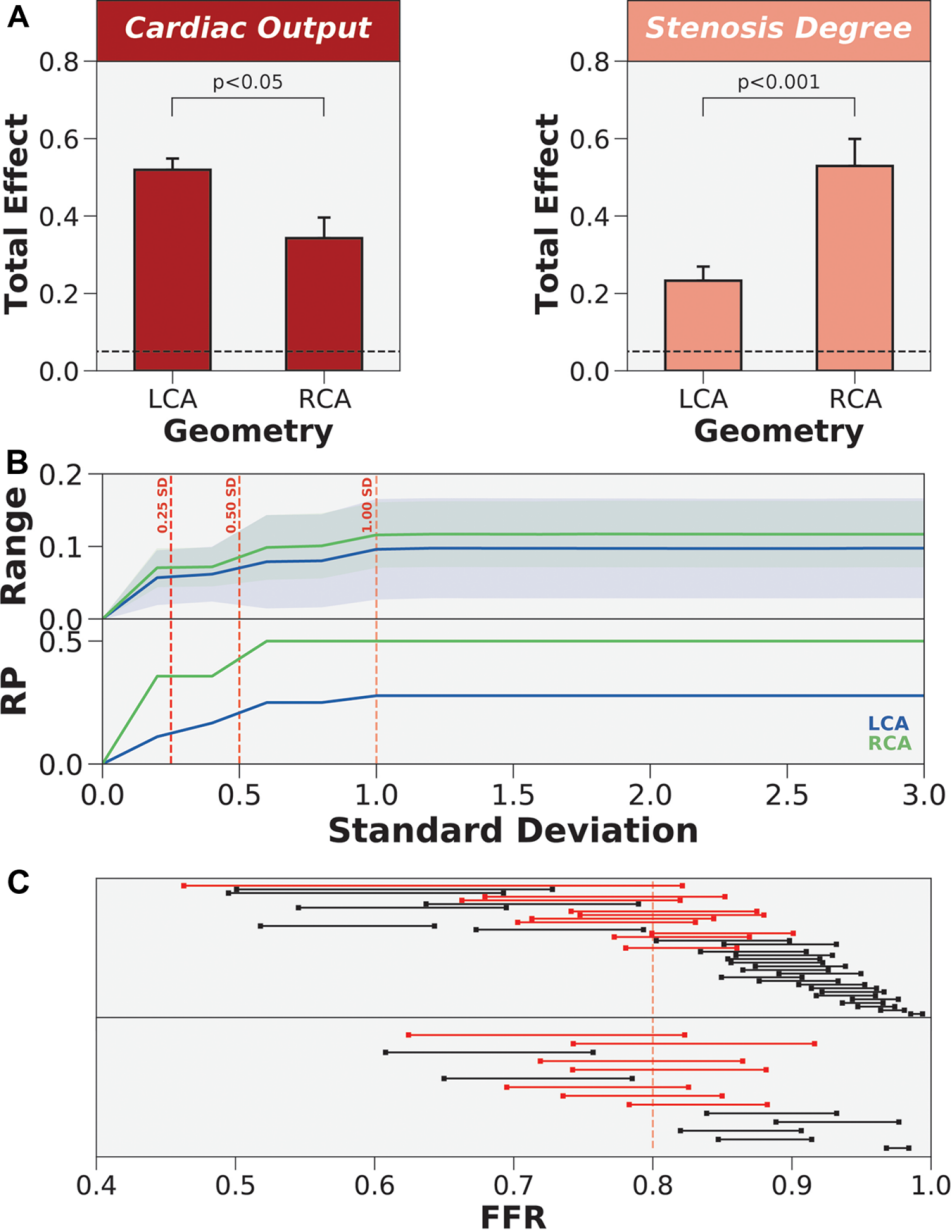
The differing impact of patient-specificity in cardiac output and stenosis degree between anatomies. (**A**) Coronary anatomy had differing effects on cardiac output and stenosis degree. LCA is left coronary artery and RCA is right coronary artery. (**B**) Re-sampling the global parameter space to estimate FFR range and reclassification proportion (RP) when only varying cardiac output and stenosis degree. Normal distributions of uncertainty were re-sampled at increasing levels of standard deviation (SD). (**C**) Dumbbell plot showing the range of FFR values when re-sampling to include 1 standard deviation of the variability in cardiac output and stenosis degree. Red dumbbells indicate re-classified cases and black dumbbells indicate cases without re-classification.

Through two-tailed t-tests (Figure 7A), the impact of patient-specificity differed between LCA and RCA for cardiac output and stenosis degree. Specifically, uncertainty in cardiac output had a larger effect on the LCA than RCA (p<0.05), and uncertainty in stenosis degree had a larger effect on the RCA than LCA (p<0.001). However, examining variances are relative metrics, and demonstrating statistically significant differences in total effects may not translate to crossing the ischemic threshold or motivating a different treatment strategy. To test if the impact of patient-specificity in cardiac output and stenosis degree could change treatment strategy, we re-sampled the normal distributions of cardiac output and stenosis degree simultaneously while restricting distal location and mean arterial pressure to their patient-specific baselines. To quantify variability in FFR, we computed an average within-patient range of FFR values across the population as a function of increasing error. Since uncertainty was modeled using normal distribution, we re-sampled the normal distributions with increasing standard deviations. The results indicated that the average range of FFR values was slightly higher in the RCA than LCA across all standard deviations (Figure 7B). We examined the proportion of the cohort that was reclassified due to uncertainty. The reclassification proportion (RP) also increased with increasing standard deviation, but demonstrated that the RCA was more sensitive to reclassification than the LCA at all standard deviation levels (Figure 7B). Both FFR range and RP curves plateaued after one standard deviation. At one standard deviation, 50% of cases were reclassified in the RCA and 25% of cases were reclassified in the LCA (Figure 7C). The patients that were reclassified were more sensitive to uncertainty and the patients that were never reclassified were less sensitive to uncertainty. In short, cardiac output and stenosis degree needed to be patient-tuned, and the impact of uncertainty could have differing effects between anatomies and patients.

## 4. Discussion

This study demonstrates the potential for CFD frameworks with minimal patient-tuned inputs to match the accuracy and diagnostic performance of frameworks with a full gamut of patient-tuned parameters, which is important because obtaining patient-specific parameters for CFD simulations is difficult and not always possible. Through global uncertainty analyses, we not only identified that stenosis degree and cardiac output (when used to parameterize peripheral resistance) were required on a per-patient level, in addition to accurately reconstructing coronary trees, but also demonstrated the validity of the findings by creating streamlined models to compare with baseline and clinical ground-truths. FFR${}_{\text{semi-streamlined}}$ had nearly-identical results with the FFR${}_{\mathrm{baseline}}$ and FFR${}_{\mathrm{streamlined}}$ was comparable in accuracy and diagnostic performance to the FFR${}_{\mathrm{baseline}}$. Furthermore, the impact of uncertainty on stenosis degree and cardiac output was shown to cause reclassification in some patients, and the impact of uncertainty had a larger effect on the RCA than LCA for stenosis degree and on the LCA than RCA for cardiac output. These results could help increase the reach and translatability of CFD frameworks for cases with missing data and scenarios when clinical data collection could be challenging.

### 4.1. FFR${}_{\mathrm{baseline}}$ compares well to categorical and continuous FFR${}_{\mathrm{invasive}}$

To create a low cost CFD framework, it was important to first demonstrate an accurate baseline high cost model compared to clinical measurements. This work used a cohort of 50 patients with a representative disease prevalence of 38% that is comparable to other studies (16, 17), and the baseline 1D FFR framework was validated against clinical ground-truths. FFR${}_{\mathrm{baseline}}$ had generally superior diagnostic performance and comparable correlation and agreement compared to computed tomography-based models (13, 60), albeit on a much smaller sample of patients. On a per-study basis, FFR${}_{\mathrm{baseline}}$ had comparable mean differences and diagnostic performance compared to other 3D or 0D coronary angiography-based models (8, 19, 20, 61–68). The studies that validate angiography-based 1D FFR is generally less than 0D or 3D models. Of note, Mohee et al. (69) validated a coronary angiography-based 1D model against invasive FFR. FFR${}_{\mathrm{baseline}}$ had better correlation and diagnostic performance. Other global uncertainty analysis studies have generally validated their models with smaller patient cohorts and use 3D models as ground-truth. Fossan et al. (27) validated their 1D model of FFR in 13 patients with 24 stenoses, but used 3D FFR models as ground-truth. Morris et al. (11) validated a pseudotransient model in 20 patients against full transient 3D CFD models. There were also studies focusing on idealized or few patient-specific geometries (26, 28, 51). In contrast, the high cost, patient-tuned, 1D model had high diagnostic performance (sensitivity=89.5%, specificity=93.6%) compared to clinical measurements of FFR and provided a robust baseline of models to investigate patient-specificity.

### 4.2. Streamlining clinical inputs maintain comparatively high diagnostic performance

To develop streamlined models, we explored how the variance contribution of clinical inputs impacted FFR. We found that cardiac output, stenosis degree, and distal location crossed the 0.05 threshold for sensitivity, and mean arterial pressure narrowly crossed the threshold. The effect size for cardiac output and stenosis degree far exceeded that of distal location. Based on the global uncertainty quantification results, we created streamlined models using only personalized values for coronary anatomy (including stenosis anatomy) and cardiac output for FFR${}_{\mathrm{streamlined}}$ and additionally with patient-specific distal location for FFR${}_{\text{semi-streamlined}}$.

The semi-streamlined model had identical diagnostic performance with the baseline and slightly better accuracy and mean difference. The streamlined model maintained high diagnostic performance (sensitivity=79.0%, specificity = 90.3%) while introducing minimal bias as compared to the baseline model (mean difference = 0.01±0.07) and clinical measurements (mean difference = 0.01±0.08). We also successfully recovered the ischemic threshold using ROC curves and demonstrated minimal bias for all three models. The semi-streamlined model had nearly identical AUC with the baseline model. This finding indicated that it was possible to use a streamlined set of inputs, and nearly identical performance could be maintained if there was a patient-specific distal location. The streamlined model had slightly lower AUC and represents a worst-case scenario, where a relatively high diagnostic performance could still be maintained without a clinically-indicated distal location.

In the streamlined framework, only two waveforms were used for the entire population, one for the LCA and one for the RCA. This indicated that patient-derived waveforms may not be needed. A common ostial diameter was used to convert coronary flow velocity to flow rates, which suggested that coronary anatomy did not matter at the inlet but mattered in terminal branches when controlling flow distribution around the coronary tree. As FFR${}_{\text{semi-streamlined}}$ almost perfectly matched FFR${}_{\mathrm{baseline}}$, mean arterial pressure, heart rate, and hematocrit did not tangibly influence FFR. The discrepancy in performance between FFR${}_{\text{semi-streamlined}}$ and FFR${}_{\mathrm{streamlined}}$ could be solely attributed to distal location. While a relatively high diagnostic performance was maintained with FFR${}_{\mathrm{streamlined}}$, the non-negligible influence of distal location on FFR was observed here and was also consistent with the global uncertainty analysis results.

We demonstrated that peripheral resistance and stenosis anatomy contributed most to the variance in FFR, and that peripheral resistance had the largest variance contribution. These results are consistent with current literature (11, 27–29) that also investigated the contribution of input parameters to FFR using global uncertainty quantification. In this study, we further identified cardiac output as the input that contributed most to peripheral resistance. We used a patient-generalized mean arterial pressure to determine peripheral resistances, which demonstrated that cardiac output was more important than mean arterial pressure in distributing flow down the coronary tree. Ultimately, the framework has the flexibility to accept a full set of patient-tuned inputs, representing the best-case scenario, but could also simulate cases with missing data using the streamlined models without compromising much diagnostic performance.

### 4.3. Impact of geometry reconstruction and cardiac output differ by coronary vessel

Coronary arteries can widely vary in anatomy and physiology (70–72), especially in diseased cases where there could be disturbed blood flow dynamics. Existing sensitivity analysis and uncertainty quantification studies assume generalizability (11, 27). We further investigated the impact of uncertainty across anatomy (LCA vs. RCA) and physiology (ischemic vs. non-ischemic identified by FFR${}_{\mathrm{invasive}}$, grey-zone vs. non-grey-zone). The grey-zone is a known cluster of FFR values where there is uncertainty on how to treat patients (59, 73). Global repeated measures ANOVA identified that the main effect of anatomy was significant and had the same effect size as grey-zone. From a variance perspective, the results indicated that the difference between grey-zone and non-grey-zone cases was comparable to the difference between LCA and RCA. Therefore, the impact of uncertainty was not generalizable across coronary geometry. There was also a significant interaction effect between anatomy and the repeated measure of total effects in clinical inputs. We first subdivided the repeated measures and found that cardiac output and stenosis degree had significant main effects, which highlighted that the impact of uncertainty was not generalizable in these clinical inputs. Conversely, distal location had no statistically significant main or interaction effects. To characterize how the impact of uncertainty varied in the LCA and RCA, we performed post hoc analyses and discovered that uncertainty in cardiac output had a larger effect on the LCA than RCA and uncertainty in stenosis degree had a larger effect on the RCA than LCA. The impact of error on FFR was not only different across anatomy, but how the effect differed also varied between clinical inputs. As total effects are relative, we also re-sampled the global parameter space to examine whether the uncertainty could cause reclassification and warrant a different treatment strategy. The parameter space was re-sampled incrementally, from the baseline inputs to 3 standard deviations of the error parameter space. These results demonstrated that the magnitude of error in cardiac output and stenosis degree was sufficient to reclassify a considerable proportion of the population. Measuring accurate clinical inputs should be prioritized on a coronary anatomy-specific level. Hence, accurate anatomic reconstruction and measurement of cardiac output were important in accurately computing FFR.

### 4.4. Clinical translatability of streamlined 1D models

It is important to consider whether the pathway to clinical translation is feasible. While models such as FFR${}_{\mathrm{angio}}$ (CathWorks, Kfar-saba, Israel) (15–17) have already paved the way for clinical translation, these state-of-the-art frameworks rely on a full gamut of patient-specific inputs for accurate FFR assessment. This work indicated that a few clinical parameters were needed, namely cardiac output and stenosis degree at the minimum, to maintain diagnostic performance as compared to the invasive gold-standard. Clinical measurements such as mean arterial pressure, heart rate, and hematocrit could be omitted. This finding could be useful in the event of missing data, which is a pervasive issue seen intra- and inter-clinic (23–25). Streamlining the clinical measurement process expands the utility of currently available techniques and may reduce the barrier for clinical translation. Further, the streamlined models have several advantages over other 1D models currently undergoing the process for clinical translation. The average computation time for the 1D framework was 10.1±4.7 min. Our calculation time was more than twice as fast as other 1D models, such as 23.9±11.2 min with Siemens cFFR (74) and 27.1±7.5 min with Toshiba CT-FFR (75). Both streamlined models also had superior diagnostic performance, correlation, and mean differences compared to the Siemens and Toshiba 1D models (75, 76). Accurate geometry segmentation was shown to be an important factor, and is typically a bottleneck even for 1D simulations (77). Applying our semi-automated algorithm, accurate coronary reconstructions were completed within 10 min (32, 78). The streamlined framework contributes to clinical translatability by identifying the measurements that could be patient-generalized and those that need to be patient-specific for accurate FFR computation.

### 4.5. Limitations

Regarding limitations, the patient population was retrospective and from a single center. A prospective study from multiple centers would provide a more robust validation, but the point of this work was to develop an optimized model from a fully patient-specific model, and validating with clinically measured FFR demonstrated that both models were accurate. Second, the correlation between FFR${}_{\mathrm{baseline}}$ and FFR${}_{\mathrm{invasive}}$ was moderate. There are multiple ways to validate FFR${}_{\mathrm{baseline}}$ against the clinical ground-truth. FFR is fundamentally a dichotomous metric used to refer patients to percutaneous coronary intervention (FFR≤0.80) or optimal medical therapy (FFR>0.80). In this work, we validated both continuous and categorical FFR. While the Bland-Altman mean differences indicated negligible bias, the correlation was moderate. FFR${}_{\mathrm{baseline}}$ was comparable to other studies in the literature. The vast majority of 1D FFR models validate against 3D models (27, 29, 52, 79–81). Compared to studies that also validated with invasive FFR (75, 76, 82), FFR${}_{\mathrm{baseline}}$ had generally higher correlation. Categorical FFR was validated using diagnostic performance metrics (i.e., sensitivity, specificity, positive predictive value, negative predictive value, accuracy, AUC) and exceeded 90% for nearly all metrics. Third, the effect of adenosine was considered generalizable across the cohort. Lo et al. (83) recently compared patient-specific outflow conditions based on myocardial perfusion from positron emission tomography data to the conventional scaling method. They found that the effect of adenosine could be overestimated and result in overestimating FFR severity. The FFR${}_{\mathrm{baseline}}$ vs. FFR${}_{\mathrm{invasive}}$ slope exceeded unity, which could indicate that our model overestimated hyperemia because of using scaling laws. This could be rectified in future works by acquiring myocardial perfusion data and tuning hyperemia on a per-patient level (83, 84). Fourth, the factorial study subdividing patient-specificity by anatomic and hemodynamic classes was limited. Although we explored coronary anatomy, ischemic vs. non-ischemic stenoses, and grey-zone cases, the study could have also explored differing effects of patient-specificity on factors such as age, sex, and the presence of co-morbidities. A larger cohort would be required to expand on the number of factors investigated. We also primarily focused on focal lesions. Including complex coronary disease, such as ostial and bifurcation stenoses, would require separate sensitivity studies as we expect hemodynamics to differ from focal stenoses. For example, ostial stenoses may have an impact on inlet coronary waveforms and bifurcation stenoses may increase the importance of segmenting accurate stenoses.

### 4.6. Conclusion

In this study, we developed two streamlined models with minimal clinical inputs that could compute FFR accurately. This work demonstrated that patient-generalized parameters could be used to accurately recover diagnostic phenomarkers and that the impact of error was not generalizable across varying anatomy and physiology. We presented a flexible framework that could enable cases with missing data to be simulated accurately. Additionally, the proposed framework could help improve the translatability and use of CFD models to guide interventional planning.

## Data Availability

The raw data supporting the conclusions of this article will be made available by the authors, without undue reservation.
