# Supplementary material for: Analysis identifying minimal governing parameters for clinically accurate in silico fractional flow reserve
Source: Front Med Technol. 2022 Dec 6;4:1034801. doi: 10.3389/fmedt.2022.1034801 (PMC9764219; doi:10.3389/fmedt.2022.1034801)
Supplement: Supplementary file 1 [file Datasheet1.pdf]

# *Supplementary Material*

## 1 SUPPLEMENTARY DATA

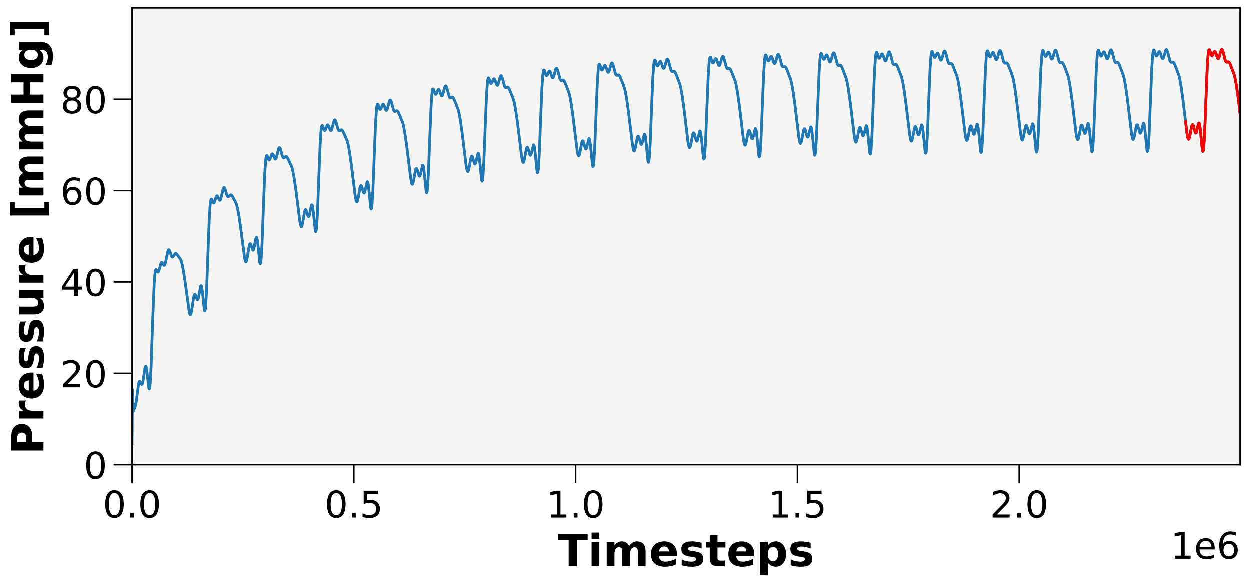


Figure S1: **Temporal convergence for time-averaged pressure at the distal location.** This is a representative case demonstrating temporal convergence. The time-average pressure was used to compute FFR. The final cardiac cycle (shown in red) was used for analysis and had an $L_2$ error of 0.0005.

**Table S1. Spatial convergence.** Spatial convergence was noted at 500 $\mu m$ for computing time-averaged pressure with $L_2$ error of 0.0003.

| Grid spacing $(mm)$ | Pressure $[mmHg]$ | $L_2$ error |
|---|---|---|
| 2.0 | 71.645 | 0.0103 |
| 1.0 | 70.804 | 0.0076 |
| 1.0 | 69.465 | 0.0010 |
| 0.5 | 68.693 | 0.0003 |

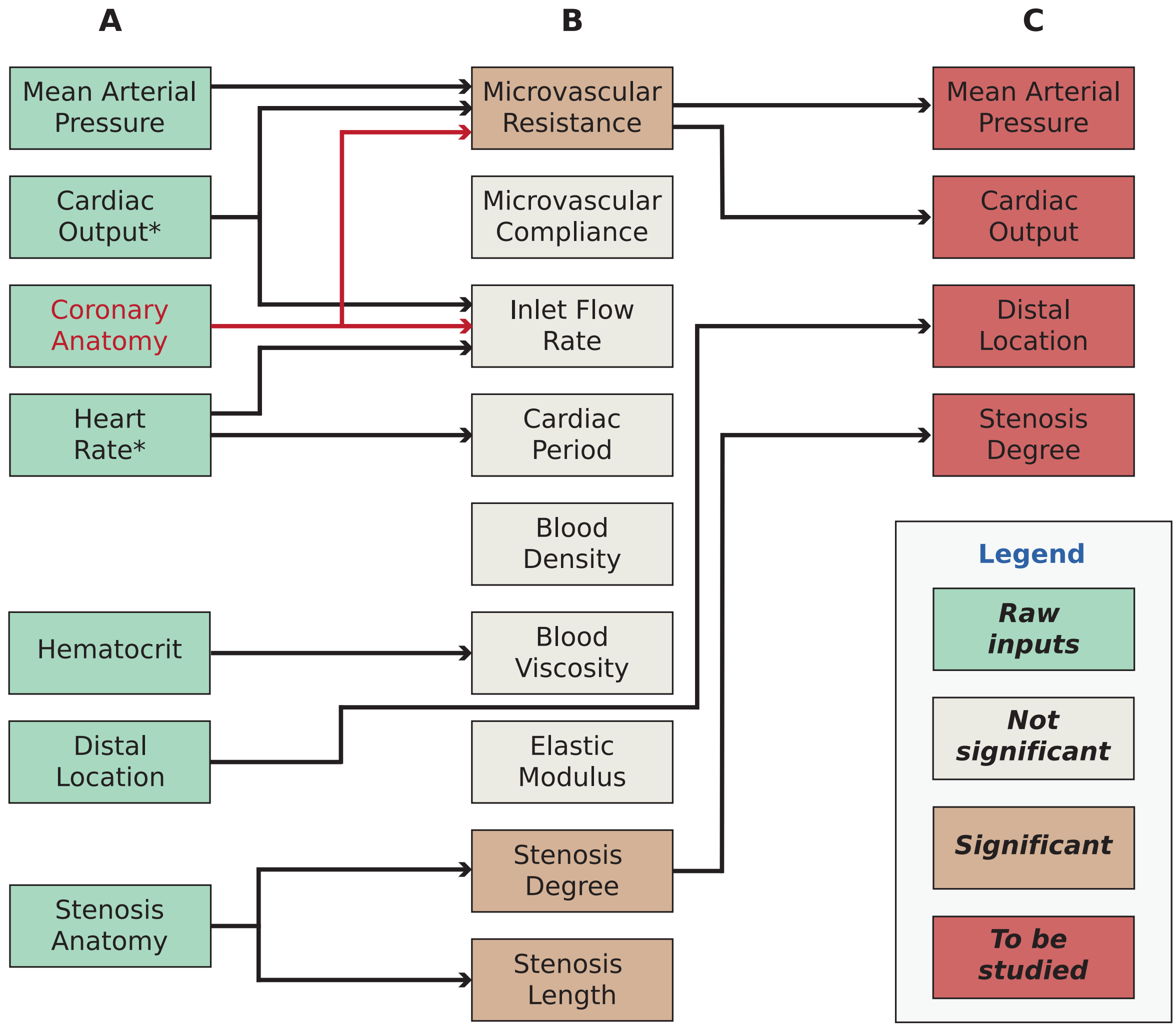


Figure S2: **Clinical inputs used to parameterize patient-specific coronary simulations.** Arrows indicate parameter dependencies and box colors label parameters as raw inputs, non-significant or significant, and inputs we investigated in this work. (**A**) The raw clinical inputs used to parameterize high cost, baseline, 1D blood flow simulations. Coronary anatomy was a necessary input to measure accurately per-patient, so this input was not perturbed in the uncertainty quantification. *Cardiac output and heart rate are directly proportional and were considered as coupled parameters. (**B**) Significant and non-significant parameters impacting FFR computation based on literature. (**C**) This set of parameters was interrogated using global uncertainty analysis and was used to guide the development of a low cost, streamlined, model with as few raw clinical inputs as possible while still maintaining accurate FFR computation.

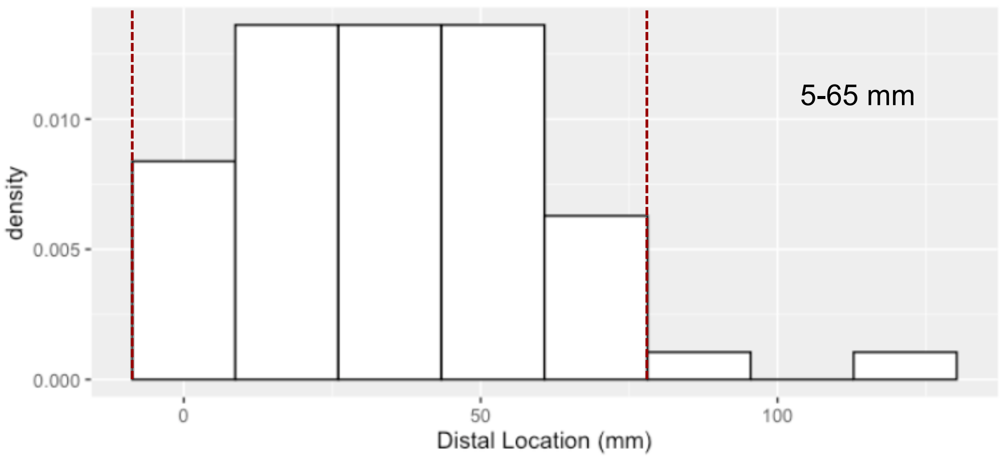


Figure S3: **Distribution of distal locations labeled by an expert interventional cardiologist for all 50 stenoses.** The 5-65 $mm$ bound was used to parameterize the uncertainty bound for distal location in the uncertainty quantification analysis.

**Table S2. Global Repeated-Measures ANOVA**. Repeated measures (RM) comprise of Sobol total effects of cardiac output, distal location, mean arterial pressure, and stenosis degree. Classification refers to stenoses above or below the ischemic threshold, grey-zone refers to patients with FFR in the 0.75-0.85 (inclusive) range or outside the range, and anatomy refers to left or right coronary arteries. p values in red indicate statistical significance.

| Between Subjects | |
|---|---|
| Classification | 0.4155 |
| Grey-zone | 0.0356 |
| Anatomy | 0.0357 |
| Classification*grey-zone | 0.5652 |
| Classification*anatomy | 0.9510 |
| Grey-zone*anatomy | 0.5160 |
| Classification*grey-zone*anatomy | 0.8733 |
| Within Subjects | |
| RM | $< 0.001$ |
| RM*classification | 0.5112 |
| RM*grey-zone | 0.6030 |
| RM*anatomy | 0.0293 |
| RM*classification*grey-zone | 0.5980 |
| RM*classification*anatomy | 0.9389 |
| RM*grey-zone*anatomy | 0.9254 |
| RM*classification*grey-zone*anatomy | 0.9995 |

**Table S3. ANOVA investigating the total effect of distal location, subdivided by classification, grey-zone, and coronary anatomy.**

| Group | p-value |
|---|---|
| Classification | 0.5779 |
| Grey-zone | 0.4579 |
| Anatomy | 0.1402 |
| Classification*grey-zone | 0.6908 |
| Classification*anatomy | 0.5596 |
| Grey-zone*anatomy | 0.5486 |
| Classification*grey-zone*anatomy | 0.9469 |

**Table S4. ANOVA investigating the total effect of cardiac output, subdivided by classification, grey-zone, and coronary anatomy.** p value in red indicates statistical significance.

| Group | p-value |
|---|---|
| Classification | 0.4034 |
| Grey-zone | 0.5723 |
| Anatomy | 0.0279 |
| Classification*grey-zone | 0.6434 |
| Classification*anatomy | 0.8388 |
| Grey-zone*anatomy | 0.9205 |
| Classification*grey-zone*anatomy | 0.9034 |

**Table S5. ANOVA investigating the total effect of stenosis degree, subdivided by classification, grey-zone, and coronary anatomy.** p value in red indicates statistical significance.

| Group | p-value |
|---|---|
| Classification | 0.2362 |
| Grey-zone | 0.9658 |
| Anatomy | 0.0025 |
| Classification*grey-zone | 0.9072 |
| Classification*anatomy | 0.7976 |
| Grey-zone*anatomy | 0.5876 |
| Classification*grey-zone*anatomy | 0.9523 |

**Table S6. ANOVA investigating the total effect of mean arterial pressure, subdivided by classification, grey-zone, and coronary anatomy.** p value in red indicates statistical significance.

| Group | p-value |
|---|---|
| Classification | 0.3698 |
| Grey-zone | 0.6389 |
| Anatomy | 0.0266 |
| Classification*grey-zone | 0.7061 |
| Classification*anatomy | 0.8215 |
| Grey-zone*anatomy | 0.9280 |
| Classification*grey-zone*anatomy | 0.9005 |
